# First Video Case Report of Chronic Retrograde Jejunojejunal Intussusception after Subtotal Gastrectomy with Braun's Anastomosis

**DOI:** 10.1155/2017/6945017

**Published:** 2017-03-07

**Authors:** Savaş Bayrak, Hasan Bektaş, Necdet Derici, Ekrem Çakar, Şükrü Çolak

**Affiliations:** ^1^Department of General Surgery, Istanbul Training and Research Hospital, Istanbul, Turkey; ^2^Private Ataköy Hospital, Istanbul, Turkey

## Abstract

Intussusception, which is seen rarely in adults, is defined as the pulling or invagination of a part of the intestine into another segment of the intestine. In this case report we present chronic retrograde jejunojejunal intussusception following gastric surgery with Braun's anastomosis in adult with video presentation. A 66-year-old woman, who had undergone gastric surgery 39 years ago and cholecystectomy 20 years ago, was admitted to our clinic with the complaints about weight loss, abdominal pain, nausea, and vomiting. Upper gastrointestinal endoscopy (UGISE) was applied, and patient was treated with surgery. This case report indicates that intussusception should be considered in the presence of clinical complaints following gastric surgery, as well as importance of endoscopy in diagnosis.

## 1. Introduction

Intussusception is defined as the pulling or invagination of a part of the intestine into another segment of the intestine. Childhood cases are often idiopathic whereas intussusception seen in adults usually has different causes including benign and malign conditions such as lipoma, submucosal fibroids, gastrointestinal stromal tumors, and adenocarcinoma. Patients can also develop intussusception in the postoperative period. In adults, the complaints related to intussusception can be acute or chronic with nonspecific findings being predominant in the latter, which is one of the major factors that delay diagnosis [1, 2].

To the best of our knowledge, this is the first video case report in the literature presenting a case of chronic retrograde jejunojejunal intussusception following gastric surgery with Braun's anastomosis.

## 2. Case Report

A 66-year-old woman was admitted to our clinic with the complaints of weight loss, abdominal pain, nausea, and vomiting. She reported that she had undergone gastric surgery 39 years earlier and cholecystectomy 20 years before. She also stated that she had consulted several physicians about this problem; however, the prescribed medication did not relieve her symptoms.

For diagnostic purposes, abdominal computed tomography (CT) and upper gastrointestinal endoscopy (UGISE) were planned. The results of UGISE revealed a large amount of bilious gastric content as well as intestinal lumen with a mass at the previous Braun's anastomotic site. As the UGISE procedure progressed, the lesion was identified as an approximately 10–15 cm jejunal segment that developed due to retrograde jejunojejunal intussusception of the efferent loop at the Braun anastomotic site. This part of the intestine was observed to have spontaneously reduced. The whole procedure was video-recorded (Video 1; see Supplementary Material available online at: https://doi.org/10.1155/2017/6945017).

Following informed consent given by the patient, the surgery was performed. Since the patient history included subtotal gastrectomy, antecolic gastrojejunostomy, and Braun's anastomosis, first, the previous anastomosis was removed and the passage was unobstructed using Roux-en-Y gastrojejunostomy (Video 2). The patient did not develop any early postoperative complications and was thus discharged on the sixth day after surgery. No problem was observed over the 5-year routine follow-up by the hospital.

## 3. Discussion

The term intussusception was first defined by John Hunter in 1789 as one part of the intestine folding into another part of the intestine. Although the first case of intussusception following gastric surgery was reported by Bozzi as early as 1914, the mechanism of this condition has not yet been clarified. However, it is believed that the process starts when a lesion in the intestinal wall or an irritant in the lumen changes the intestinal peristalsis [3–5].

Intussusception is classified as “antegrade” if it is directed towards the physiological peristalsis and “retrograde” if the movement is in the opposite direction [6]. It is mostly seen in newborns and children with only 5% of all cases belonging to adults. In adult intussusception cases, 70–90% have organic causes, of which more than half are malignant [7, 8]. Intussusception is rare in the postoperative period with an incidence of less than 0.1% [2].

In the literature, five forms of intussusception are described: Type 1 is the antegrade intussusception of the afferent loop and is commonly seen with an incidence of 5.5%. Type 2, the most frequent form (75.5%), is further divided into two as follows: Type 2a, which are the retrograde intussusceptions of the efferent loop, and Type 2b efferent-efferent intussusceptions. Type 3 is the combination of the first two types and is seen in 6.5% of patients. The last form, Type 4, is the intussusception at the site of Braun's anastomosis and has an 8% incidence [9]. As can be seen in Video 2, the case presented here was identified as Type 4 characterized by chronic retrograde jejunojejunal intussusception at the anastomotic site. We think that the reader is better able to understand the subject with visual materials. This makes the article different from other similar manuscripts.

In the literature, several factors have been reported to contribute to the development of intussusception, including adhesions in the suture line, making long intestinal loops during surgery, increased intra-abdominal pressure, reverse peristalsis, and short jejunal mesentery [3]. In the present case, no mechanical or functional cause was identified. The acute form presents with symptoms associated with a high level of intestinal obstruction including severe epigastric pain, sensitivity, nausea, vomiting, hematemesis, and palpable masses. These complaints may lead to serious complications such as strangulation and incarceration. In the presence of these symptoms particularly in those patients with a history of gastric surgery, an intussusception diagnosis should be considered. The treatment for intussusception is emergency surgery. Although the chronic form of the condition has similar symptoms in essence, it has certain differences in terms of the symptoms being recurrent, long-term, and spontaneous with less clamorous manifestations [5]. In the present case, gastric complaints began following gastric surgery and increased significantly within the previous 3-4 years.

Since intussusception is rarely seen in adults, it is difficult to diagnose. Therefore, given the presence of clinical suspicion, further examination is necessary with UGISE, ultrasonography (US), barium swallow tests, and abdominal CT being conventionally used in the diagnosis of intussusception [10]. In the present study, the diagnosis was made following endoscopy. Since there is no single treatment option that can be used for all patients, the chosen method should be specific to the individual. In the literature, a frequently recommended method is the reduction or resection of the affected part of the intestine to unblock it, closing previous anastomoses and performing reanastomosis. However, Ozdogan et al. suggested that, in cases presenting with normal intestinal viability, manual reduction is sufficient [11].

To date, several methods have been proposed to prevent the recurrence of intussusception following treatment, including but not limited to suturing the jejunum or mesentery to the neighboring tissue, fixing the afferent loop mesentery to the efferent loop and converting Billroth II to Billroth I [12]. The complaints of the present case had persisted for a long time; therefore, resection Roux-en-Y gastrojejunostomy was performed on the patient. Since no complication was observed in the early postoperative period, the patient was discharged on the sixth day after surgery.

## 4. Conclusion

Intussusception should be considered in the presence of clinical complaints following gastric surgery and diagnosed using appropriate diagnostic tools to reduce the morbidity and mortality associated with this condition.

## Supplementary Material

Upper gastrointestinal endoscopy and surgery procedure was given video1. 2.
